# Characterization of a NRPS-like Protein from *Pestalotiopsis fici* for Aldehyde Generation

**DOI:** 10.3390/jof8101001

**Published:** 2022-09-23

**Authors:** Yuanyuan Li, Peng-Lin Wei, Huomiao Ran, Jie Fan, Wen-Bing Yin

**Affiliations:** 1 State Key Laboratory of Mycology, Institute of Microbiology, Chinese Academy of Sciences, Beijing 100101, China; 2 Savaid Medical School, University of Chinese Academy of Sciences, Beijing 100049, China

**Keywords:** NRPS-like enzyme, fungal carboxylate reductase (CAR), aldehyde generation, benzoic acid derivatives, *Pestalotiopsis fici*

## Abstract

Nonribosomal peptide synthetase (NRPS)-like enzymes containing A-T-R domain architecture are also known as carboxylate reductases (CARs) for aldehyde generation. To identify new members of CARs, we established a virtual library containing 84 fungal CARs distributed in seven distinct clades by genome mining and phylogenetic analysis. Nine CARs, including PnlA from *Pestalotiopsis fici* and eight known CARs, were clustered in clade VI and proposed to catalyze the reduction of nonreducing polyketide synthase (NR-PKS)-derived aryl carboxylic acids. The recombinant protein PnlA was overproduced and purified to apparent homogeneity from *Saccharomyces cerevisiae*. In vitro enzyme assays of PnlA with 28 different benzoic acid derivatives (**1**–**28**) revealed the corresponding aldehyde formation in 14 cases (**1**–**14**). Comparison of conversion yields indicated the high preference of PnlA toward 3,5-dimethylorsellinic acid (DMOA, **4**) and vanillic acid (**10**). A specificity-conferring code Q355 in PnlA was postulated by sequence alignment with the known CARs in clade VI. Our study provides an updated virtual library of fungal CAR enzymes and expands the biocatalytic selectivity of CARs.

## 1. Introduction

**Figure 1:**
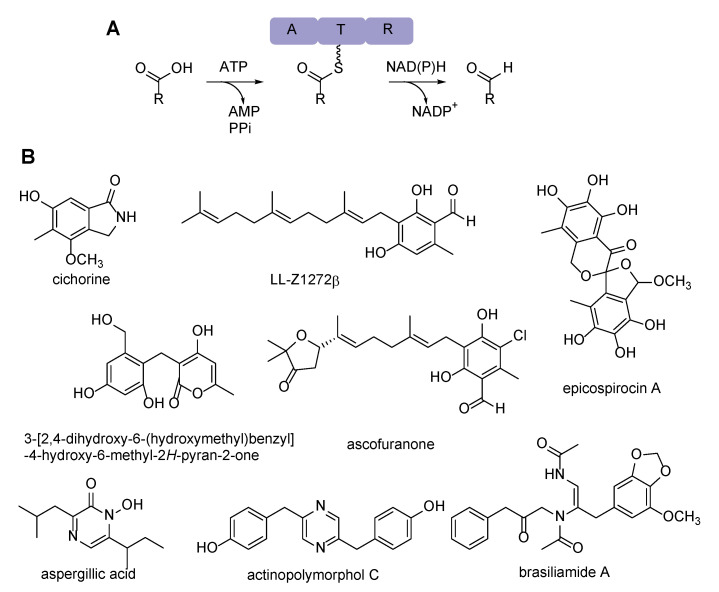
Carboxylic acid reduction by CARs. (**A**) CARs containing A-T-R domain architecture catalyze two-electron reduction of carboxylates to aldehydes. (**B**) Representative fungal NPs involving carboxylic acid reduction by CARs in their biosynthetic pathways. They include cichorine from *Aspergillus nidulans* [19], LL-Z1272b from *Stachybotrys bisbyi* [13], epicospirocin A from *Epicoccum nigrum* [14], melanin derivative from *Ustilago maydis* [20], ascofuranone from *Acremonium egyptiacum* [21], aspergillic acid from *Aspergillus flavus* [22], actinopolymorphol C from *A. flavus* [15] and brasilliamide A from *Penicillium brasilianum* [16].

Nonribosomal peptide synthetases (NRPSs) are multidomain enzymes involved in the biosynthesis of structurally and functionally diverse nonribosomal peptides (NRPs) [1]. Examples of NRPs used clinically include vancomycin, daptomycin, penicillin, cephalosporins and ergotamine [2,3]. In general, the minimal catalytic module for peptide extension comprises adenylation (A), thiolation (T), also termed peptidyl carrier protein (PCP), as well as condensation (C) domains. However, the peptide-forming C domain can be replaced by a thioesterase (TE) or reductase (R) domain, constructing two subfamilies of NRPS-like enzymes [4,5]. Recent advances in genome sequencing have indicated the widespread presence of NRPS-like genes in microorganisms, whereas only a small number were characterized for secondary metabolism. Represented by ApvA, BtyA and AtqA identified from *Aspergillus terreus*, NRPS-like enzymes with an A-T-TE domain architecture catalyze the dimerization of α-keto acids, leading to the formation of lactones, ketals or benzoquinones [6,7]. Differing from the TE domain, the R domain in NRPS-like enzymes functions for carboxylic acid reduction to afford aldehyde with the help of upstream substrate adenylation by the A domain at the expense of ATP, thioester linkage formation by T domain and NADPH as a hydride donor (Figure 1A) [8]. Hence, NRPS-like enzymes containing A-T-R domain architecture are also known as carboxylate reductases (CARs).

On the basis of characterization of the first CAR, NcCAR, from the fungus *Neurospora crassa*, CARs triggered research interest owing to the remarkable roles of aldehyde-containing compounds as biologically active agents and key synthetic intermediates [9,10,11]. However, only approximately 50 CARs were identified from microorganisms and produced recombinantly in *Escherichia coli* or *Saccharomyces cerevisiae* for biocatalytic investigations. CARs have shown considerable potential for product diversification, as A domains of CARs are differentiated to accept a broad range of substrates, including aryl carboxylic acids, aliphatic carboxylic acids, aryl-aliphatic carboxylic acids and amino acids [12]. Therefore, CARs have been connected to various types of natural products (NPs), such as the meroterpenoid LL-Z1272β [13], spiroketal epicospirocin A [14], as well as piperazine derivatives actinopolymorphol C and brasiliamide A [15,16] (Figure 1B). Exemplified by the well-studied AtCAR (ATEG_03630) from *A. terreus*, TvCAR from *Trametes versicolor* and NcCAR, CARs exhibited relaxed catalytic activities toward a variety of carboxylic acids [9,17,18]. This renders CARs ideal biocatalysts for aldehyde synthesis. However, the selective specificities of CARs have remained unclear until now.

**Scheme 1:**
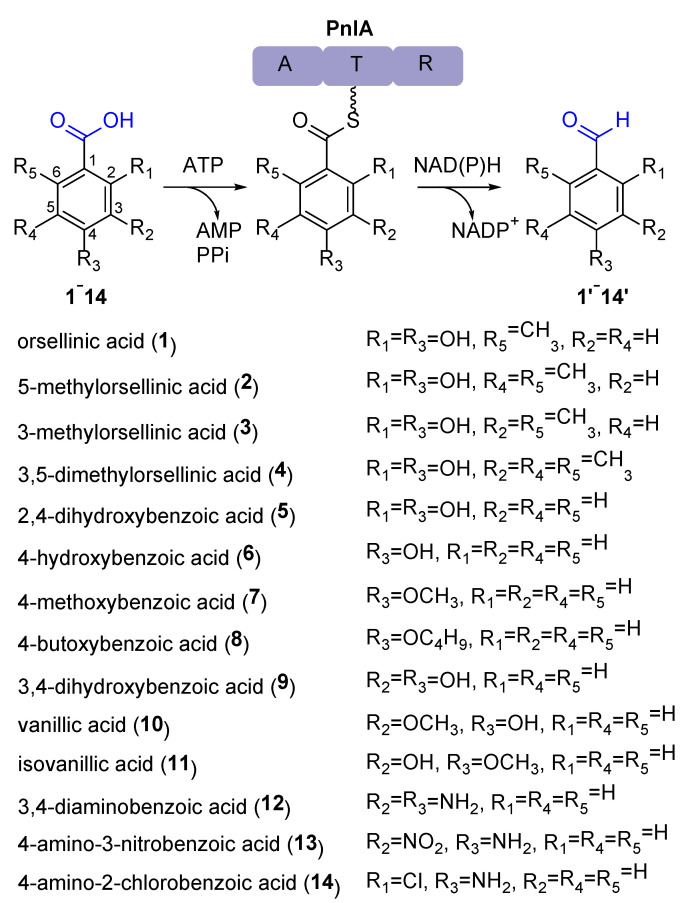
Mechanism of the PnlA-catalyzed reduction of aryl carboxylic acid derivatives. Fourteen aryl carboxylic acid derivatives (**1**–**14**) were converted to the corresponding aldehydes (**1′**–**14′**).

The biocatalytic potential of CARs encouraged us to identify additional new members for aldehyde generation. We conducted genome mining of CARs in fungi and established a fungal virtual CAR library containing 84 fungal CARs with 55 new CARs. These CARs were clustered into seven distinct clades by phylogenetic analysis. A new CAR, termed PnlA, was identified from *Pestalotiopsis fici* by recombinant production and in vitro biochemical investigation. PnlA showed relaxed catalytic activities toward 14 aryl carboxylic acid derivatives but with varying levels of preferences (Scheme 1). Combined with multiple sequence alignments, we proposed a key specificity-conferring code of PnlA.

## 2. Materials and Methods

### 2.1. Strain, Media and Culture Conditions

The strains used in this study are summarized in Table S1. *P. fici* CGMCC3.15140 was grown on potato dextrose agar (PDA, BD Difco™, 39 g/L) or potato dextrose broth (PDB, BD Difco™, 24 g/L) at 25 °C [23,24]. *Escherichia coli* strain DH5α was cultured in an LB medium (*w*/*v*, 1% NaCl, 1% tryptone and 0.5% yeast extract) with appropriate antibiotics for standard DNA manipulation [25]. *Saccharomyces cerevisiae* strain BJ5464-NpgA (MATα *ura3*-52 *his3*-D200 *leu2*-D1 *trp1 pep4*::HIS3 *prb1* D1.6R *can1* GAL) was incubated on YPD medium (*w*/*v*, 2% peptone, 1% yeast extract and 2% dextrose) for routine growth and on SD-drop medium (*w*/*v*, 0.5% Bacto™ Casamino Acids, 0.17% yeast nitrogen base, 0.5% ammonium sulfate and 2% dextrose) with appropriate nutrition as required for gene heterologous expression at 30 °C [26,27]. The substrates used in this study were purchased from Beijing Hua Wei Rui Ke Chemical Co., Ltd. (Beijing, China), Beijing Wokai Biotechnology Co., Ltd. (Beijing, China), Beijing Bailingwei Technology Co., Ltd. (Beijing, China) and Shanghai Yuanye Biotechnology Co., Ltd. (Shanghai, China).

### 2.2. Computer-Assisted Sequence Analysis

Using the known NcCAR and AtCAR as templates, we searched the CAR protein sequences from the NCBI database (http://www.ncbi.nlm.nih.gov/protein, accessed on 15 August 2022) and presented 84 CARs from 28 fungal species. All amino acid sequences of CARs were aligned by ClustalW using MEGA 7.0 for cluster analysis. The phylogenetic tree was constructed based on maximum likelihood (ML) analysis using RAxML 8.2.10 and visualized in iTOL v6 (https://itol.embl.de/, accessed on 20 August 2022). Multiple sequence alignments were performed with the program ClustalW and visualized with ESPript 3.2 (http://espript.ibcp.fr/ESPript/cgi-bin/ESPript.cgi, accessed on 20 August 2022) to identify strictly conserved amino acid residues.

### 2.3. RNA Isolation and cDNA Synthesis

For isolation of RNA from *P. fici*, the fungus was cultivated on PDA at 25 °C for 7 days. Total RNA from the mycelia of *P. fici* was extracted by the TranZol™ kit (Transgen Biotech, Beijing, China) [28]. RNA integrity was confirmed by electrophoresis on the Tris-boric acid-EDTA (TBE) agarose gel and a Quawell Q3000 nucleotide analyzer (Quawell). The single-strand cDNA was synthesized by a FastKing RT kit (Tiangen Biotech, Beijing, China) according to the manufacture’s protocol.

### 2.4. Plasmid Construction

The primers and plasmids are given in Table S1. For PnlA protein production under *ADH2* promoter (*ADH2p*) in *S. cerevisiae*, TransStart ^®^ FastPfu DNA polymerase (Transgene Biotech, Telangana, India) was used for amplification. The PCR mixture comprised 10 μL 5 × TransStart ^®^ FastPfu buffer, 0.2 mM dNTPs, 0.2 μM forward/reverse primers PFICI364-XW55 F/R, 1 μL *P. fici* cDNA, 2.5 units TransStart ^®^ FastPfu DNA polymerase and ddH_2_O in a final volume of 50 μL. PCR was performed on a T100™ thermal cycler (Bio-Rad, Hercules, CA, USA) as follows: 95 °C for 2 min; 35 cycles of 95 °C for 20 s, 57 °C for 20 s and 72 °C for 2 min; followed by 72 °C for 5 min. The amplified fragment was purified with a DNA clean and concentrator kit (Zymo Research, Irvine, CA, USA) and cloned into the vector pXW55 with Q5 ultra-fidelity DNA polymerase (New England Biolabs, Ipswich, MA, USA) using a quick-change method [29,30], generating pYLYY7 (Figure S1). The plasmid pYLYY7 was confirmed by enzyme validation and sequencing (Sangon Biotech, Shanghai, China).

### 2.5. Protein Overproduction and Purification, as Well as Enzyme Assays

The plasmid pYLYY7 was transformed into *S. cerevisiae* BJ5464-NpgA for PnlA protein overproduction. Yeast-competent cell preparation and transformation were performed with an S.c. Easy comp^TM^ transformation kit (Invitrogen, Carlsbad, CA, USA) according to the manufacture’s protocol. *S. cerevisiae* transformants were grown in 10 mL of SC-Ura dropout media for 2 days and inoculated to 3.2 L of YPD medium. Cells were grown at 30 °C and 220 rpm for 3 days. The cells were harvested by centrifugation (4000 rpm, 8 min, 4 °C), resuspended in 20 mL lysis buffer (50 mM NaH_2_PO_4_, 150 mM NaCl and 10 mM imidazole; pH 8.0) and lysed by SPEX freezer mill (SPEX SamplePrep, Metuchen, USA). The recombinant *His_6_*-tagged PnlA was purified by Ni-NTA affinity chromatography (Qiagen, Hilden, Germany) according to the published procedures [31]. The purified recombinant protein was concentrated into a storage buffer (50 mM Tris−HCl, 100 mM NaCl, pH = 7.9) containing 10% glycerol and analyzed on SDS-PAGE.

To determine the activity of PnlA, the enzyme assays (100 μL) containing 50 mM Tris-HCl (pH 8.5), 10 mM MgCl_2_, 1 mM substrate, 10 mM ATP, 2 mM NADPH, 0.7–6% (*v*/*v*) glycerol, up to 5% (*v*/*v*) DMSO and 35 μg of purified PnlA were carried out at 30 °C for 12 h. The reaction mixtures were terminated by extraction with 200 μL ethyl acetate twice and evaporated under reduced pressure to afford crude extracts. Before injecting to liquid chromatography–mass spectrometry (LC-MS), the crude extracts were dissolved in methanol and centrifuged at 13,000 rpm for 15 min. Conversion yields of the enzyme reactions were calculated from peak areas of aldehyde products and aryl carboxylic acid substrates as analyzed on LC-MS.

### 2.6. LC-MS Analysis

LC-MS analysis was performed on an Agilent HPLC 1200 series system equipped with a single-quadrupole mass-selective detector and an Agilent 1100LC MSD model G1946D mass spectrometer using a Venusil XBP C18 column (3.0 by 50 mm, 3 μm, Bonna-Agela Technologies, Tianjin, China). Water (A) with 0.1% (*v*/*v*) formic acid and acetonitrile (B) were used as the solvents at a flow rate of 0.5 mL/min. The substances were eluted with a linear gradient from 2% to 30% (*v*/*v*) solvent B in 30 min, then washed with 100% (*v*/*v*) solvent B for 5 min and equilibrated with 2% (*v*/*v*) solvent B for 5 min. The mass spectrometer was set in electrospray positive-ion mode for ionization.

## 3. Results

### 3.1. Construction of a Fungal Virtual CAR Library

**Figure 2:**
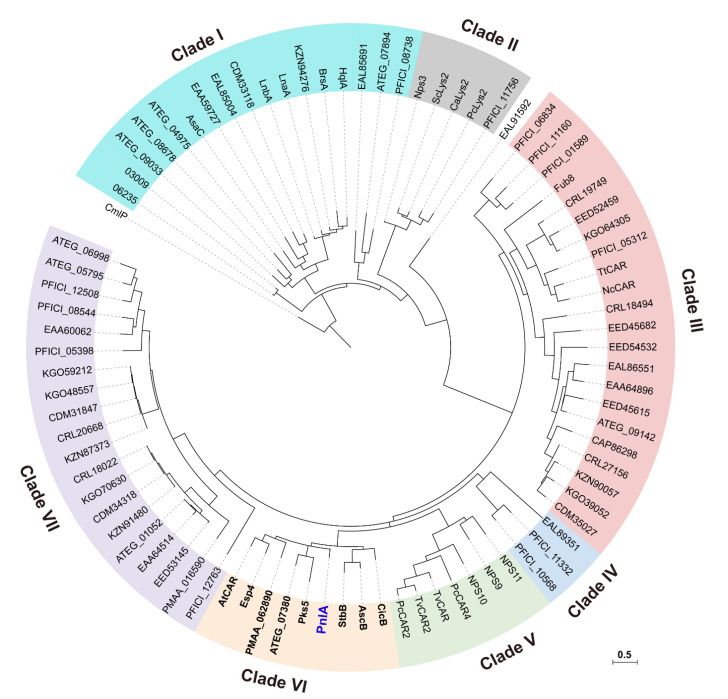
Phylogenetic analysis of 84 CAR protein sequences from fungi. Seven distinct clades are shown in different colors. CARs in clade I, II and VI are mainly responsible for the reduction of amino acids, L-aminoadipate and orsellinic acid derivatives, respectively. CmlP characterized in *Streptomyces venezuelae* was used as the outgroup to root the tree [38]. Multiple alignments were performed by ClustalW with MEGA 7.0. The phylogenetic tree was constructed based on maximum likelihood (ML) analysis using RAxML 8.2.10 and visualized in iTOL v6. Amino acid sequences were obtained from the GenBank database.

To identify new members, we searched for fungal CARs using NcCAR and AtCAR as templates through the BLASTP program [9,17]. A total of 84 proteins from 28 fungal species were revealed as CARs with the typical A-T-R domain architecture (Figure 2). 29 of them have been previously reported for carboxylic acid reduction by genome mining and in vitro enzyme assays [4,8,32]. Ten CARs containing a well-known AtCAR and nine other CARs were mined in *A. terreus*, which is consistent with a previous study [4]. The genome of *A. flavus* harbors eight CAR-encoding genes with three known CARs, LnaA, LnbA and AsaC, accepting L-Tyr, Leu or Ile as substrates [15,22]. Four to six CARs were first found in the genomes of four *Penicillium* species, including *P. camemberti*, *P. chrysogenum*, *P. expansum* and *P. roqueforti*, whereas none of them was connected to the secondary metabolite (SM) biosynthesis. With respect to an endophytic fungus, *P. fici*, an A-T-R domain structure was observed in thirteen enzymes, including PFICI_01589, PFICI_00364, PFICI_05312, PFICI_05398, PFICI_06834, PFICI_08544, PFICI_08738, PFICI_10568, PFICI_11160, PFICI_11332, PFICI_11756, PFICI_12508 and PFICI_12763. However, neither biosynthetic nor biochemical investigation was reported for these thirteen CARs [23,28].

Hence, we carried out a sequence comparison among these 84 CARs by phylogenetic analysis, leading to the construction of a CAR similarity network (Figure 2). Seven distinct clades were observed, with three relatively large clades containing 17 (clade I), 22 (clade III) and 20 sequences (clade VII), respectively. On the basis of the strict specificities of 03009 and 06235 for Ala/Val/Ser [33], AsaC for Leu/Ile [22], LnaA, LnbA and HqlA for L-Tyr [15,34] and BrsA for L-Phe [16], we proposed that the CARs clustered in clade I were involved in amino acid reduction. Represented by Nps3 and Lys2, CARs in clade II are L-aminoadipate reductases [35]. In clade III, only NcCAR and TtCAR were reported with relaxed substrate tolerances for reducing aryl-aliphatic carboxylic acids, aliphatic carboxylic acids and a few heterocyclic aromatic carboxylic acids [8]. Fub8 from *Fusarium fujikuroi* catalyzes the reduction of *trans*-2-hexenoic acid in the biosynthesis of fusaric acid [36]. Differing from other clades, CARs in clade V are mainly identified from basidiomycetes [18,33]. In addition, no CAR was known in clades IV and VII until now.

A newly identified PFICI_00364, hereafter termed PnlA, was found in clade VI, together with eight other known CARs, including StbB, AscB, CicB, Esp4, AtCAR, PMAA_062890, ATEG_07380 and Pks5 [13,14,17,19,20,21,37]. Interestingly, the eight CARs shared similarities on their catalytic preferences for the aryl carboxylic acid derivatives, such as orsellinic acid (OA, **1**), 5-methylorsellinic acid (5-MOA, **2**) and 3-methylorsellinic acid (**3**). Pks5 from *Ustilago maydis* reduced **1** to produce orsellinaldehyde (**1′**), undergoing melanin biosynthesis [20]. StbB and AscB showed activity toward grifolic acid, a meroterpenoid derived from **1** [13,21]. AtCAR, CicB and Esp4 catalyzed the carboxylic acid reduction of **2**, but only AtCAR was characterized for its broad substrate scope [14,17,19,37].

### 3.2. Overproduction and In Vitro Characterization of PnlA

**Figure 3:**
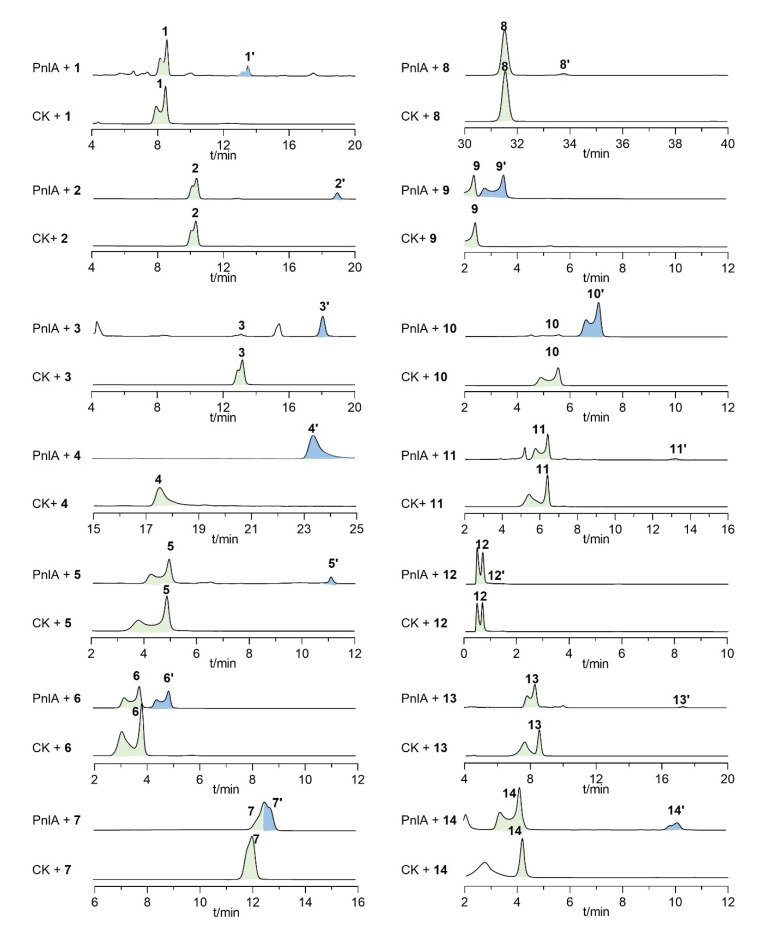
LC-MS analyses of the incubation mixtures of PnlA with different aryl carboxylic acid derivatives (**1**–**14**). **1′–14′** are the corresponding aldehyde products. UV absorptions at 280 nm are illustrated for the enzyme assays of PnlA with **1–13**, and those at 340 nm are illustrated for the enzyme assay of PnlA with **14**. CK is the reaction mixture with denatured PnlA. Additional data on the enzyme assays of PnlA with **15–28** are presented in Figure S2.

Encouraged by the reported biocatalytic activities of CARs in clade VI, we proceeded to identify PnlA by in vitro biochemical investigation. PnlA comprising three exons of 3261 bp was amplified from cDNA of P. fici, cloned into pXW55 under ADH2p and overexpressed in *S. cerevisiae* BJ5464-NpgA cells (Figure S1) [26]. Purification with the aid of Ni-NTA agarose resin resulted in 2.7 mg of recombinant PnlA per liter of standard culture. Consistent with its expected mass of 122.1 kDa, the C-terminal His_6_-tagged protein was confirmed on SDS-PAGE (Figure S1). To determine the activity of PnlA, we first incubated it with one of the most well-accepted substrates by clade VI members, **1**, in the presence of ATP, NADPH and MgCl_2_. LC-MS analysis of the incubation mixture showed a new peak at the retention time of 12.5 min in comparison with the negative control containg denatured enzyme (Figure 3). The product peak was identified as the corresponding aldehyde (**1′**) due to its [M + H]^+^ ions at m/z 153.1, which was 16 Da less than the carboxylic acid-containing **1** (Scheme 1 and Table 1). This result confirmed the newly characterized PnlA as a CAR for aryl carboxylic acid reduction.

The CAR encoding gene *pnlA* was located together with a transcription factor (TF), PFICI_00365, and a non-reducing polyketide synthase (NR-PKS), PFICI_00366, in the biosynthetic gene cluster (BGC) (Figure S3). The assembly combination of NR-PKS and CAR was also found for other CAR members in clade VI [13,14,17,19,20,21,37]. In contrast, PFICI_00366, containing a domain structure of a starter-unit acyltransferase (SAT), a ketosynthase (KS), an acyltransferase (AT), a product template (PT), an acyl carrier protein (ACP), a methyltransferase (MT) and a TE [39], shares relatively high identities of 34–37% with AusA, PrhL, AdrD, Trt4 and AndM on the amino acid level, all of which catalyze the formation of 3,5-dimethylorsellinic acid (**4**) [40,41,42,43,44]. Thus, we proposed that PFICI_00366 also catalyzes the formation of **4** as an NR-PKS assembly line, which was subsequently reduced by PnlA for aldehyde (**4′**) formation (Figure S3). To prove the activity of PnlA toward **4**, we carried out an in vitro enzyme assay in analogy to that toward **1**. Surprisingly, **4** was completely converted to the corresponding aldehyde product, **4′**, with [M + H]^+^ ions at *m*/*z* 181.0 (Figure 3 and Table 1). The remarkable conversion yield indicated that the NR-PKS product of PFICI_00366 **4** could be the natural substrate of PnlA. To the best of our knowledge, no CAR was previously discovered to accept aryl carboxylic acid containing three methyl substituents, implying the catalytic potential of PnlA to study the effect of trimethylation on substrate specificity.

### 3.3. Flexible Biocatalytic Activities of PnlA

Given the activities of PnlA toward its probably natural substrate **4** and another well-accepted substrate by CARs, **1**, we speculated that PnlA also showed flexible biocatalytic activities toward a variety of substrates. Therefore, we performed enzyme assays of PnlA with 26 different carboxylic acid derivatives (**2**, **3** and **5**–**28**) (Figure S4). LC-MS analysis of the incubation mixtures showed that PnlA accepted 12 substrates (**2**, **3** and **5**–**14**) for the corresponding aldehyde generation (**2′**, **3′** and **5′**–**14′**) (Figure 3). In contrast to AtCAR with the best accepted substrate, **2**, the activity of PnlA toward **2** dramatically decreased by ~80% in comparison with that toward **4**. However, the removal of the 5-methyl group in **4** (**3**) led to a reduced effect of the substrate specificity, with a decreasing conversion from 100% to 58.8%. In addition, only 22.6% of **1** and 16.3% of 2,4-dihydroxybenzoic acid (**5**) were consumed by PnlA. The biocatalytic activities of PnlA toward **1**–**5** indicated the importance of dimethylation at positions C3 and C5 in contributing the substrate specificity of the A domain.

When the 2-hydroxyl moiety was removed from **5**, the specific activity of PnlA toward 4-hydroxybenzoic acid (**6**) obviously increased, with a conversion yield of 45.0%. The methoxylation at the C4 position (**7**) led to a slightly lower conversion yield comparing with that with **6**. However, the activity of PnlA significantly decreased by 10-fold in the presence of larger substitution at the C4 position, like the butoxy moiety (**8**). This might be caused by the steric hindrance. In comparison with **6**, dihydroxylation at the C3 and C4 positions slightly increased the activity of PnlA (53.9%). Interestingly, PnlA showed much better acceptance for vanillic acid (**10**), with a conversion yield of 95.8%, which was only lower than the acceptance for its natural substrate, **4**, whereas only 7.7% of isovanillic acid (**11**) was consumed by PnlA. In contrast to the 4-hydroxyl-containing benzoic acids as mentioned above, no substrate consumption was observed for benzoic acid (**15**), salicylic acid (**16**) and 3-hydroxybenzoic acid (**17**) (Figure S2). These results indicate the crucial role of 4-hydroxyl substituent in benzoic acid for the substrate specificity because hydroxyl moiety interacted with the key residues of the A domain to form hydrogen bonds.

PnlA can accept 3,4-diaminobenzoic acid (**12**) and 4-amino-3-nitrobenzoic acid (**13**) to form the corresponding aldehydes according to LC-MS analysis but afford products only with trace amounts (Figure 3 and Table 1). In comparison, better acceptance was observed for 4-amino-2-chlorobenzoic acid (**14**), with a conversion yield of 22.6%, proving the importance of 4-hydroxyl substitution in benzoic acid for PnlA specificity. In addition, no aldehyde product was detected for the substrates including nitrobenzoic acids (**18**–**20**), phthalic acid (**21**), cinnamic acid derivatives (**22**–**25**) and other aryl-aliphatic carboxylic acids (**26**–**28**) (Figure S2).

**Figure 4:**
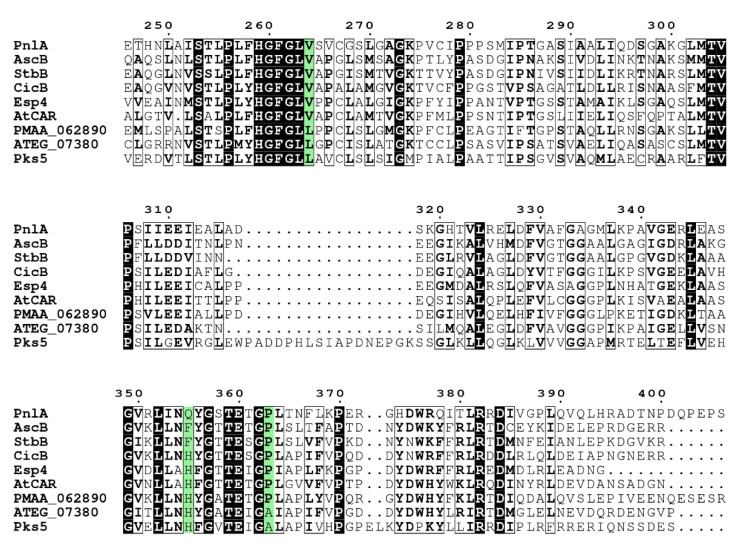
Alignment of fungal CARs located in clade VI, including PnlA, StbB [13], AscB [21], CicB [19], Esp4 [14], AtCAR [17], PMAA_062890, ATEG_07380 and Pks5 [20]. See alignment of the whole sequences in Figure S5.

Considering the differing substrate specificities of the CARs in clade VI, we further carried out the multiple sequence alignments (Figure 4 and Figure S5). The conserved residues of CARs can be found in PnlA, such as G260, V264, T304, L324 and L352, which were also described for AtCAR. However, residue differences at the 355 position were revealed, e.g., Q355 in PnlA, F355 in StbB and AscB, as well as H355 in six other proteins. Previous studies indicated H355 as a key residue of AtCAR for recognizing **2** as its predominant substrate. This explains its high conservation in Esp4 and CicB, with the substrate preference of **2 [14,19]**. In contrast, StbB and AscB contained F355 at this site, which might be relevant to their preference for the prenylated substrate [13,21]. Thus, we elucidated Q355 as a crucial residue for the A domain of PnlA in the catalytic process.

## 4. Discussion

Aldehyde-containing products, such as vanillin, benzaldehyde and cinnamaldehyde, have attracted the attention of researchers owing to their importance in the fragrance and flavoring industries and their high reactivities via nucleophilic addition [32,45]. Chemical reduction strategies of carboxylic acids normally result in low yields of aldehyde formation and require the introduction of protecting groups to limit over-reduction or off-target reduction. Alternatively, biocatalytic strategies, including carboxylic acid reduction and alcohol oxidation, can efficiently provide the targeted aldehyde products [10]. Enzymatic reduction of carboxylic acids via CARs was reported as earlier as 1959 for NcCAR characterization [9]. Genome mining and the techniques of genetic manipulation have led to approximately 50 identified CARs with a broad substrate scope for accepting aromatic, polycyclic and heteroaromatic carboxylic acids; aliphatic carboxylic acids; aryl-aliphatic carboxylic acids; and amino acids [8]. Inspired by the catalytic potential of CARs, we constructed a fungal virtual CAR library containing 84 enzymes with 55 new CARs from 28 species for the first time in this study.

The combination of phylogenetic analysis of 84 fungal CARs and enzyme/substrate correlations of 29 known CARs prompted us to postulate the substrate scopes of fungal CARs (Figure 2). For example, 17 CARs clustered in clade I represent a toolbox for catalysis of amino acid reduction, probably undergoing piperazine core formation as the products of LnaA, LnaB, HqlA and BrsA [15,16,34]. Nine CARs clustered in clade VI, including a newly identified PnlA from *P. fici* and eight other known CARs, demonstrating substrate preferences for orsellinic acid derivatives, which were assembled by NR-PKS. An NR-PKS-encoding gene, PFICI_00366, was also found in the same cluster with *pnlA*. Therefore, we proposed PFICI_00366 for assembly of **4** based on the its high identities with the reported biosynthetic enzymes and the subsequent reduction to generate its aldehyde product (**4′**) (Figure S3) [40,41,42,43,44]. Notably, the conversion of **4** to **4′** by a CAR was identified for the first time in this study, to the best of our knowledge. These results imply a strategy to generate aldehyde products by coexpression of an NR-PKS gene with a CAR gene.

In analogy to AtCAR, in vitro biochemical investigation of PnlA also revealed a broad substrate scope for accepting 14 of 28 benzoic acid derivatives (Figure 3 and S4). **4** and **10** are the best accepted substrates of PnlA, with conversion yields of 100% and 95.8%, respectively (Table 1). The comparison of the substrate consumption indicated the importance of 4-hydroxyl substitution in benzoic acid for the substrate specificity of PnlA, as this moiety functioned in the interaction with the A domain to construct hydrogen bonds. Furthermore, we postulated a key residue, Q355 of PnlA, contributing to its biocatalytic specificity by multiple sequence alignments. In contrast, eight other CARs in clade VI carry F355 and H355, which might determine their substrate preferences for **1** and **2** or the prenylated **1**. Although cinnamic acid (**22**) is one of the most popular CAR substrates in previous studies, no consumption of **22** or its derivatives (**23**–**25**) can be observed in the incubation mixtures with PnlA.

In summary, we established a fungal virtual CAR library containing 84 fungal CARs with 55 new CARs. Their substrate scopes can be postulated on the basis of phylogenetic analysis and the known CAR substrate scopes. The recombinant protein production in *S. cerevisiae* and in vitro biochemical investigation promoted us to identify PnlA from *P. fici* as a new CAR for aldehyde generation. We proved the flexible biocatalytic activities of PnlA toward 14 benzoic acid derivatives (**1**–**14**) and uncovered its specificity for 4-hydroxylated benzoic acids. A key specificity-conferring code, Q355, was postulated for the A domain of PnlA. Our study provides a promising biocatalyst for aldehyde generation.

## Figures and Tables

**Table 1:** Specific activities of PnlA toward different benzoic acid derivatives.

Substrate	[M + H]^+^ of PnlA Product		Conversion
	Calculated	Measured	
orsellinic acid (**1**)	153.1	153.1	22.6%
5-methylorsellinic acid (5-MOA, **2**)	167.1	167.0	19.2%
3-methylorsellinic acid (3-MOA, **3**)	167.1	166.9	58.8%
3,5-dimethylorsellinic acid (DMOA, **4**)	181.1	181.0	100.0%
2,4-dihydroxybenzoic acid (**5**)	139.0	139.3	16.3%
4-hydroxybenzoic acid (**6**)	123.0	122.9	45.0%
4-methoxybenzoic acid (**7**)	137.1	137.0	40.0%
4-butoxybenzoic acid (**8**)	179.1	179.0	4.1%
3,4-dihydroxybenzoic acid (**9**)	139.0	139.0	53.9%
vanillic acid (**10**)	153.1	152.9	95.8%
isovanillic acid (**11**)	153.1	152.9	7.7%
3,4-diaminobenzoic acid (**12**)	137.1	136.8	t.a.
4-amino-3-nitrobenzoic acid (**13**)	167.0	166.8	6.3%
4-amino-2-chlorobenzoic acid (**14**)	156.0	156.0	22.6%
benzoic acid (**15**)	107.0	n.d.	0
salicylic acid (**16**)	123.0	n.d.	0
3-hydroxybenzoic acid (**17**)	123.0	n.d.	0
2-nitrobenzoic acid (**18**)	152.0	n.d.	0
3-nitrobenzoic acid (**19**)	152.0	n.d.	0
4-nitrobenzoic acid (**20**)	152.0	n.d.	0
phthalic acid (**21**)	151.0 or 135.0	n.d.	0
cinnamic acid (**22**)	133.1	n.d.	0
coumaric acid (**23**)	149.1	n.d.	0
caffeic acid (**24**)	165.1	n.d.	0
ferulic acid (**25**)	179.1	n.d.	0
4-hydroxyphenylacetic acid (**26**)	137.1	n.d.	0
2-hydroxyphenylacetic acid (**27**)	137.1	n.d.	0
4-aminophenylacetic acid (**28**)	136.1	n.d.	0

t.a., trace amount; the conversion yield was less than 1%; n.d., not detected by LC-MS.

## Data Availability

The data presented in this study are available in this manuscript and can be requested from the corresponding author.

## Supplementary Materials

The following supporting information can be downloaded at: https://www.mdpi.com/article/10.3390/jof8101001/s1, Table S1: Plasmids, strains and primers; Figure S1: Overproduction of PnlA in *S. cerevisiae*; Figure S2: LC-MS analyses of the incubation mixtures of PnlA with different benzoic acid derivatives (**15**–**28**), but no corresponding aldehyde product was observed; Figure S3: The biosynthetic gene cluster (BGC) containing *pnlA* and its proposed biosynthetic pathway; Figure S4: All substrates screened for PnlA biocatalytic activity in this study; Figure S5: Alignment of fungal CARs located in clade VI, including PnlA, StbB, AscB, CicB, Esp4, AtCAR, PMAA_062890, ATEG_07380 and Pks5.
