# Supplementary material for: Characterization of a NRPS-like Protein from Pestalotiopsis fici for Aldehyde Generation
Source: J Fungi (Basel). 2022 Sep 23;8(10):1001. doi: 10.3390/jof8101001 (PMC9605436; doi:10.3390/jof8101001)
Supplement: Supplementary file 1 [file jof-08-01001-s001.zip › jof-1926934-supplementary.pdf]

Supplementary Materials for

Characterization of an NRPS-Like Protein from *Pestalotiopsis fici* for Aldehyde Generation

Yuanyuan Li [1,2,†], Peng-Lin Wei [1,2,†], Huomiao Ran [1], Jie Fan [1,*] and Wen-Bing Yin [1,2,*]

[1] State Key Laboratory of Mycology, Institute of Microbiology, Chinese Academy of Sciences, Beijing 100101, China
[2] Savaid Medical School, University of Chinese Academy of Sciences, Beijing 100049, China

* Correspondence: fanjie@im.ac.cn (J.F.); yinwb@im.ac.cn (W.-B.Y.); Tel.: +86-010-64806170 (W.-B.Y.)
† These authors contributed equally to this work.

Table of Contents

Supplementary tables **3**

Table S1. Plasmids, strains and primers 3

Supplementary figures 4

Figure S1. Overproduction of PnlA in *S. cerevisiae* 4

Figure S2. LC-MS analyses of the incubation mixtures of PnlA with different benzoic acid derivatives (**15–28**), but no corresponding aldehyde product was observed.. 5

Figure S3. The biosynthetic gene cluster (BGC) containing *pnlA* and its proposed biosynthetic pathway 6

Figure S4. All substrates screened for PnlA biocatalytic activity in this study 7

Figure S5. Alignment of fungal CARs located in clade VI, including PnlA, StbB, AscB, CicB, Esp4, AtCAR, PMAA_062890, ATEG_07380 and Pks5 11

References 12

Supplementary tables

Table S1. Plasmids, strains and primers

**Strains/Plasmids**	**Description**
Pestalotiopsis fici CGMC3.15140	Wild type [1]
Escherichia coli DH5α	[2]
Saccharomyces cerevisiae BJ5464-NpgA	[3]
pXW55	*2μ, URA3, ADH2p::ACPC, Amp*^{r} [4]
pYLYY7	pXW55-PnlA; a 3,320 bp fragment of *pnlA* from cDNA of *P. fici* inserted in pXW55

**Primers**	**Sequence 5' to 3'**	**Targeted amplification**
PFICI364-XW55 F	gattataaggatgatgatgataagactagtatgacatcattcgttccgcc	a 3,320 bp fragment containing the coding region of *pnlA* from *P.fici* cDNA and the homologous arms to construct pYLYY7
PFICI364-XW55 R	aattagtgatggtgatggtgatgcacgtgtgcaaacaattggatcttcttcca	
PnlA RT-F	ggcttgatgactgtgccatc	a 1,581 bp partial fragment of *pnlA* from cDNA of *P. fici* to validate the plasmid of pYLYY7
PnlA RT-R	ggcaccgtaggactgtagg	

pXX = plasmid

Supplementary figures

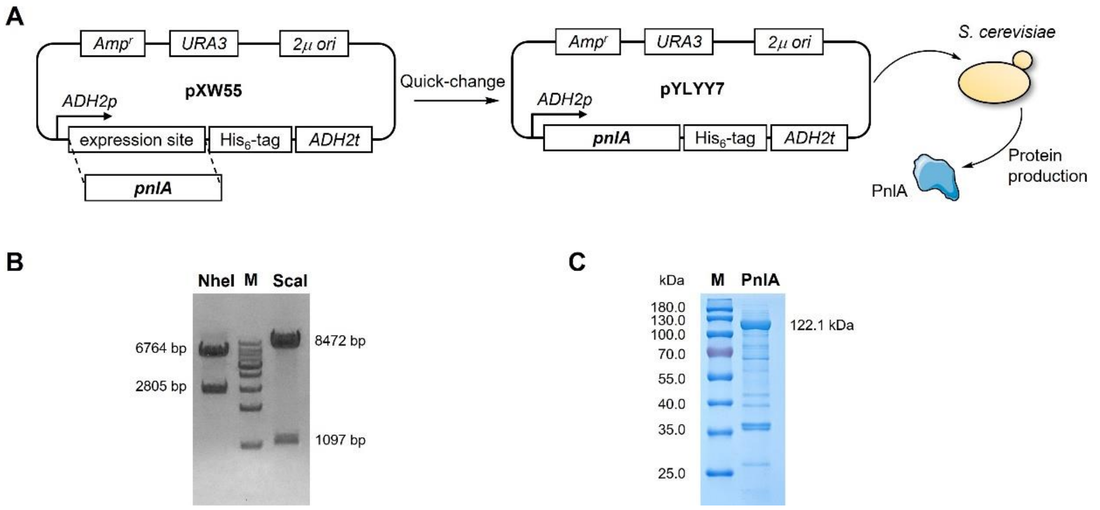

Figure S1. Overproduction of PnlA in *S. cerevisiae*. (A) The strategy of *pnlA* overexpression in *S. cerevisiae*. (B) The enzyme validation of pYLYY7 harboring *pnlA* in pXW55 under *ADH2p* by NheI and ScaI. (C) Analysis of the purified PnlA on SDS-PAGE.

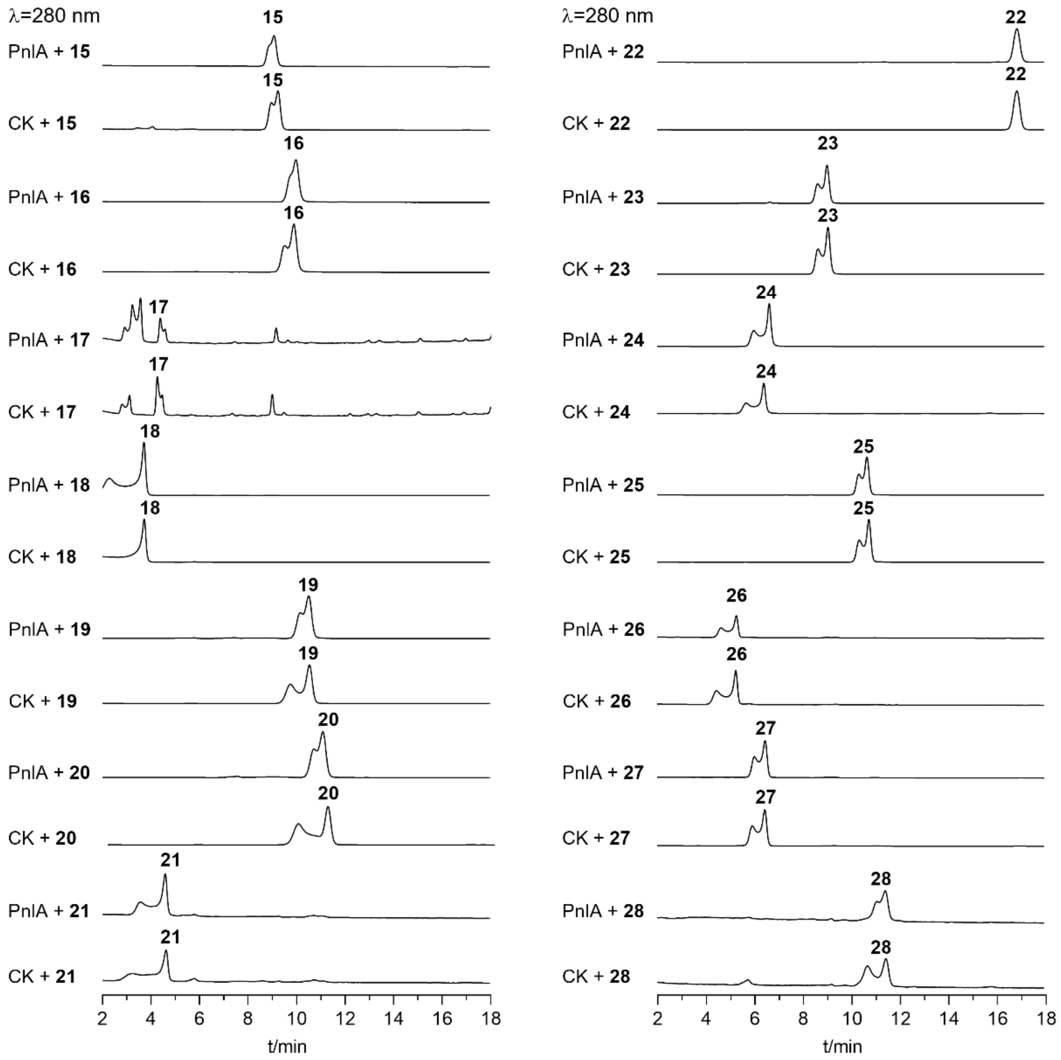

Figure S2. LC-MS analyses of the incubation mixtures of PnlA with different benzoic acid derivatives (**15–28**), but no corresponding aldehyde product was observed. UV absorptions at 280 nm are illustrated. CK, the reaction mixture with denatured PnlA. See more data for the enzyme assays of PnlA with **1–14** in figure 3.

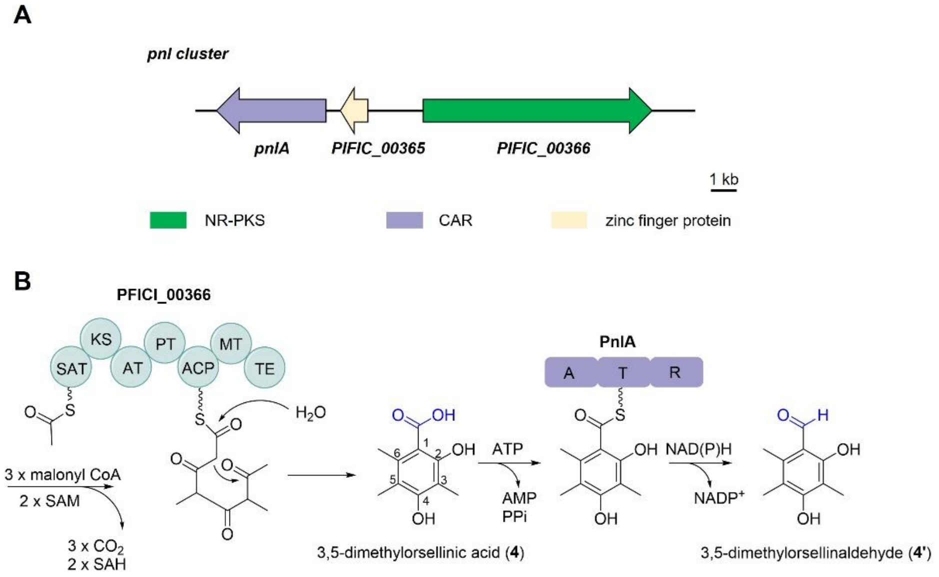

Figure S3. The biosynthetic gene cluster (BGC) containing *pnlA* and its proposed biosynthetic pathway. (A) *pnl* cluster in *P. fici* containing a NRPS-like (also termed carboxylic acid reductase (CAR)) gene *pnlA*, a transcription factor PFICI_00365 and a non-reducing (NR)-PKS PFICI_00366. (B) The NR-PKS PFICI_00366 catalyzes the formation of 3,5-dimethylorsellinic acid (**4**), undergoing the reduction by PnlA to give 3,5-dimethylorsellinaldehyde (**4'**). SAT, starter-unit acyltransferase; KS, ketosynthase; AT, acyltransferase; PT, product template; ACP, acyl carrier protein; MT, methyltransferase; TE, thioesterase; R, reductase.

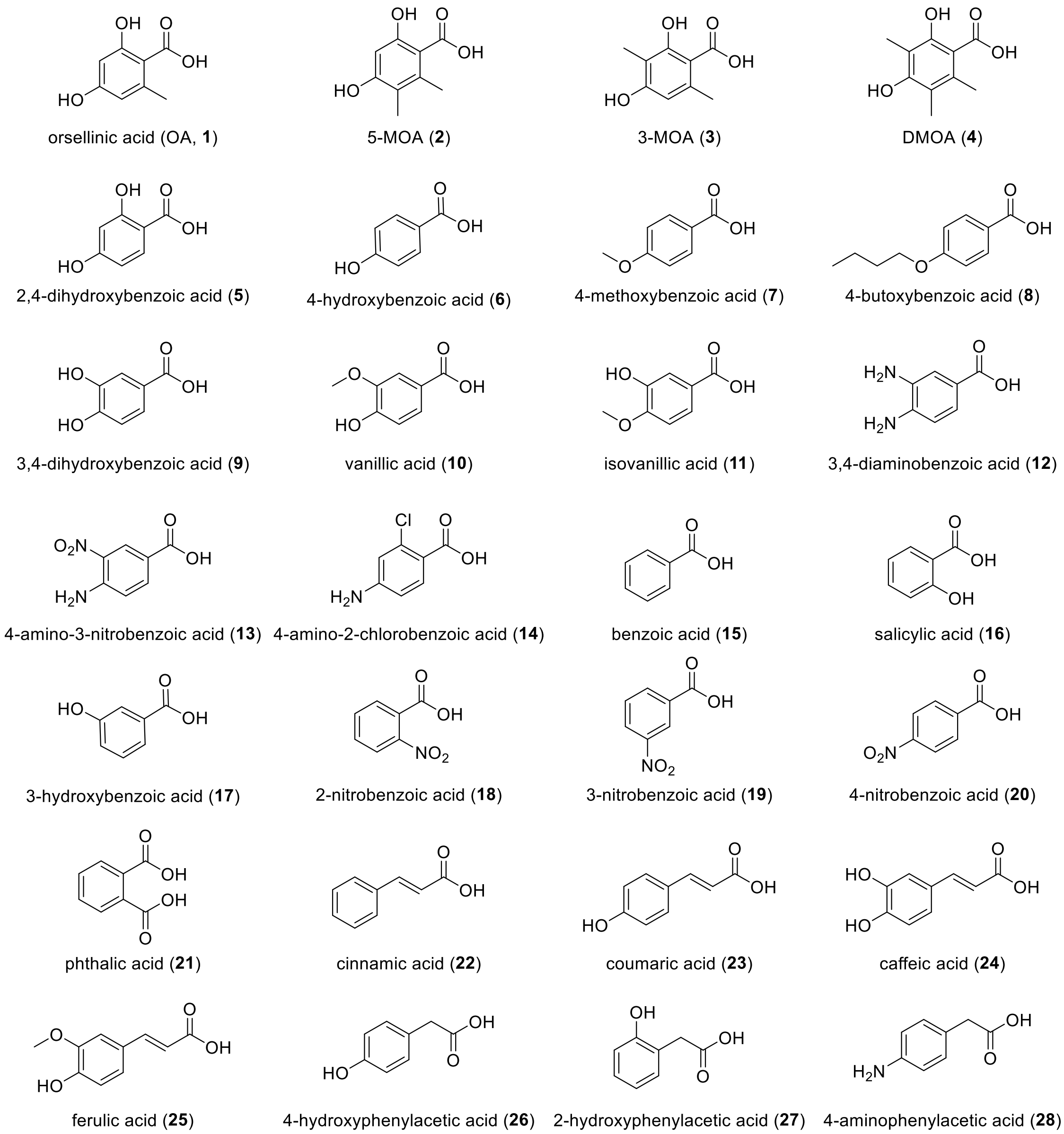

Figure S4. All substrates screened for PnlA biocatalytic activity in this study. They include orsellinic acid (OA, **1**), 5-methylorsellinic acid (5-MOA, **2**), 3-methylorsellinic acid (3-MOA, **3**), 3,5-dimethylorsellinic acid (DMOA, **4**), 2,4-dihydroxybenzoic acid (**5**), 4-hydroxybenzoic acid (**6**), 4-methoxybenzoic acid (**7**), 4-butoxybenzoic acid (**8**), 3,4-dihydroxybenzoic acid (**9**), vanillic acid (**10**), isovanillic acid (**11**), 3,4-diaminobenzoic acid (**12**), 4-amino-3-nitrobenzoic acid (**13**), 4-amino-2-chlorobenzoic acid (**14**), benzoic acid (**15**), salicylic acid (**16**), 3-hydroxybenzoic acid (**17**), 2-nitrobenzoic acid (**18**), 3-nitrobenzoic acid (**19**), 4-nitrobenzoic acid (**20**), phthalic acid (**21**), cinnamic acid (**22**), coumaric acid (**23**), caffeic acid (**24**), ferulic acid (**25**), 4-hydroxyphenylacetic acid (**26**), 2-hydroxyphenylacetic acid (**27**) and 4-aminophenylacetic acid (**28**).

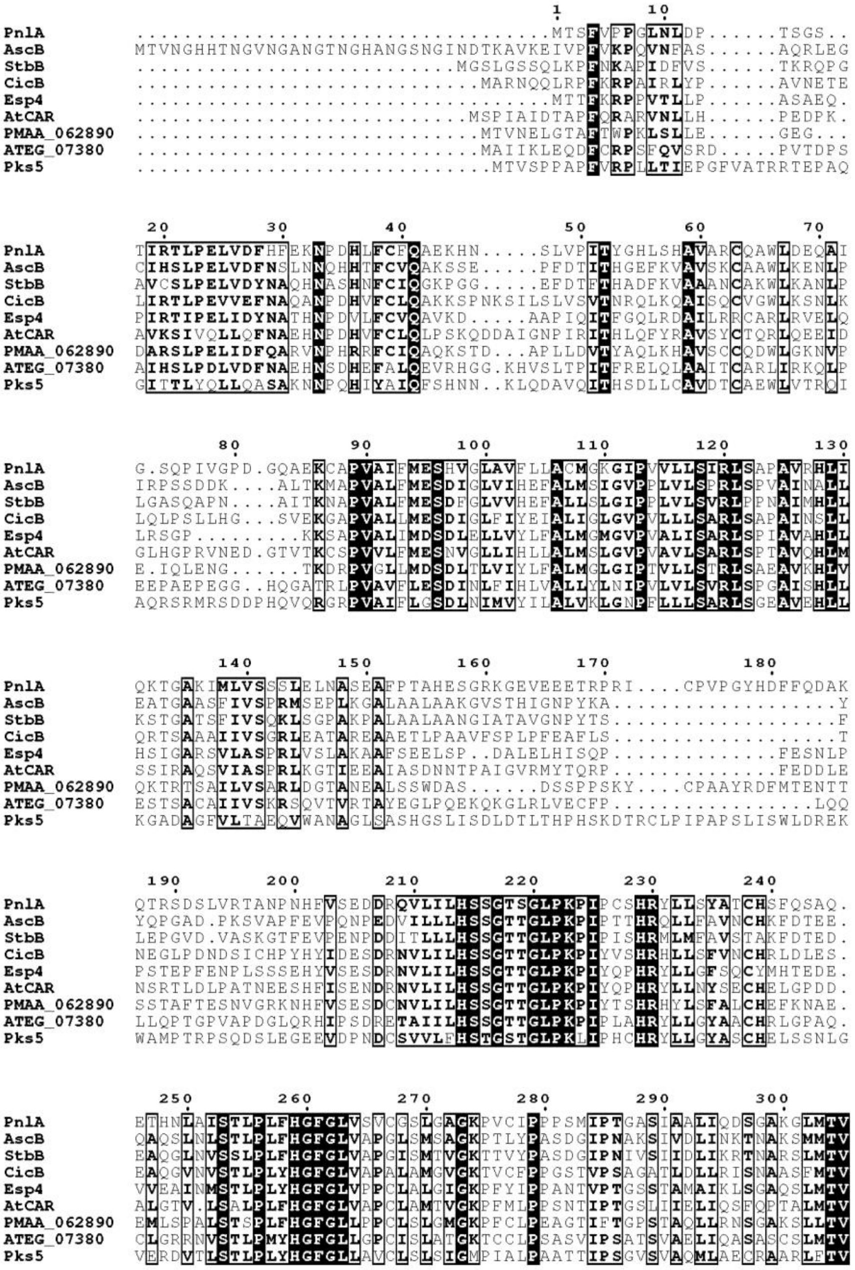

(*continued*)

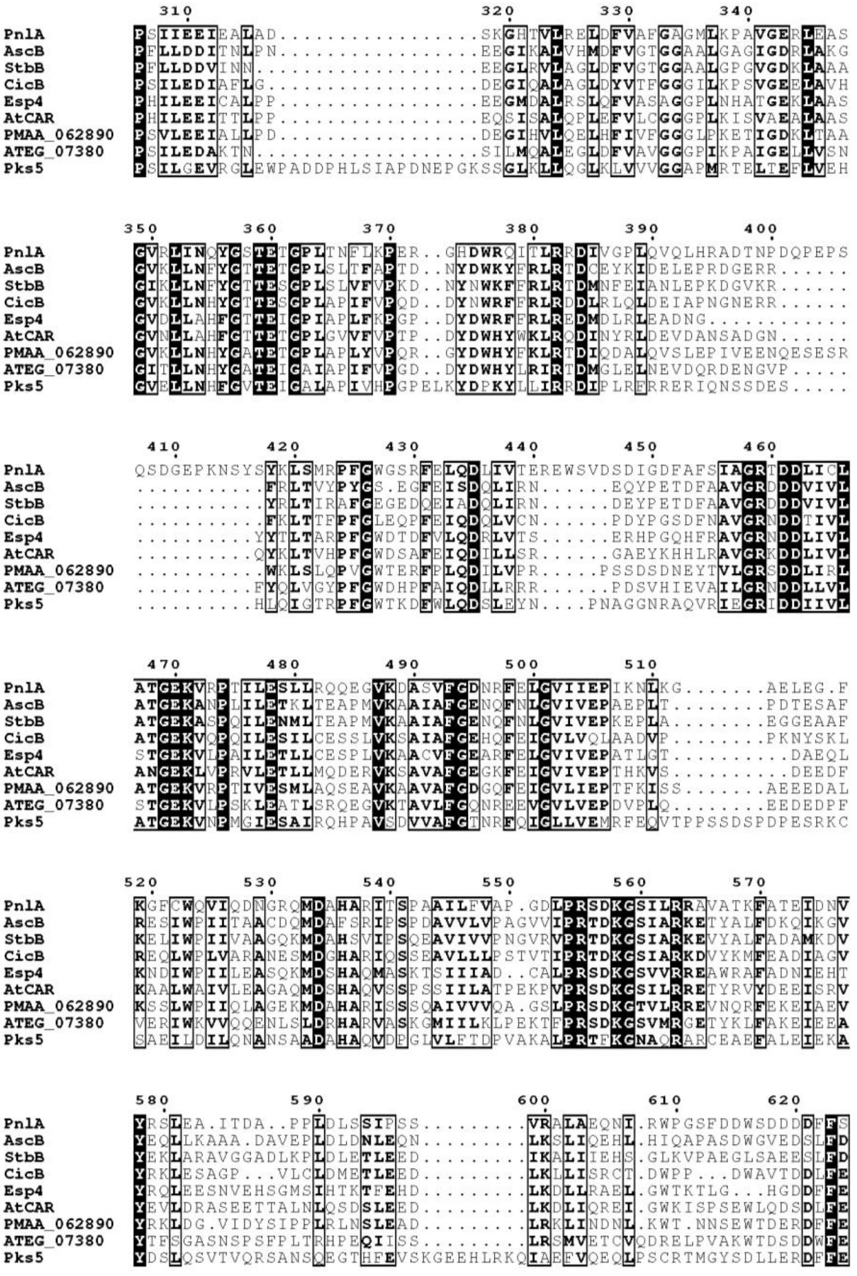

(*continued*)

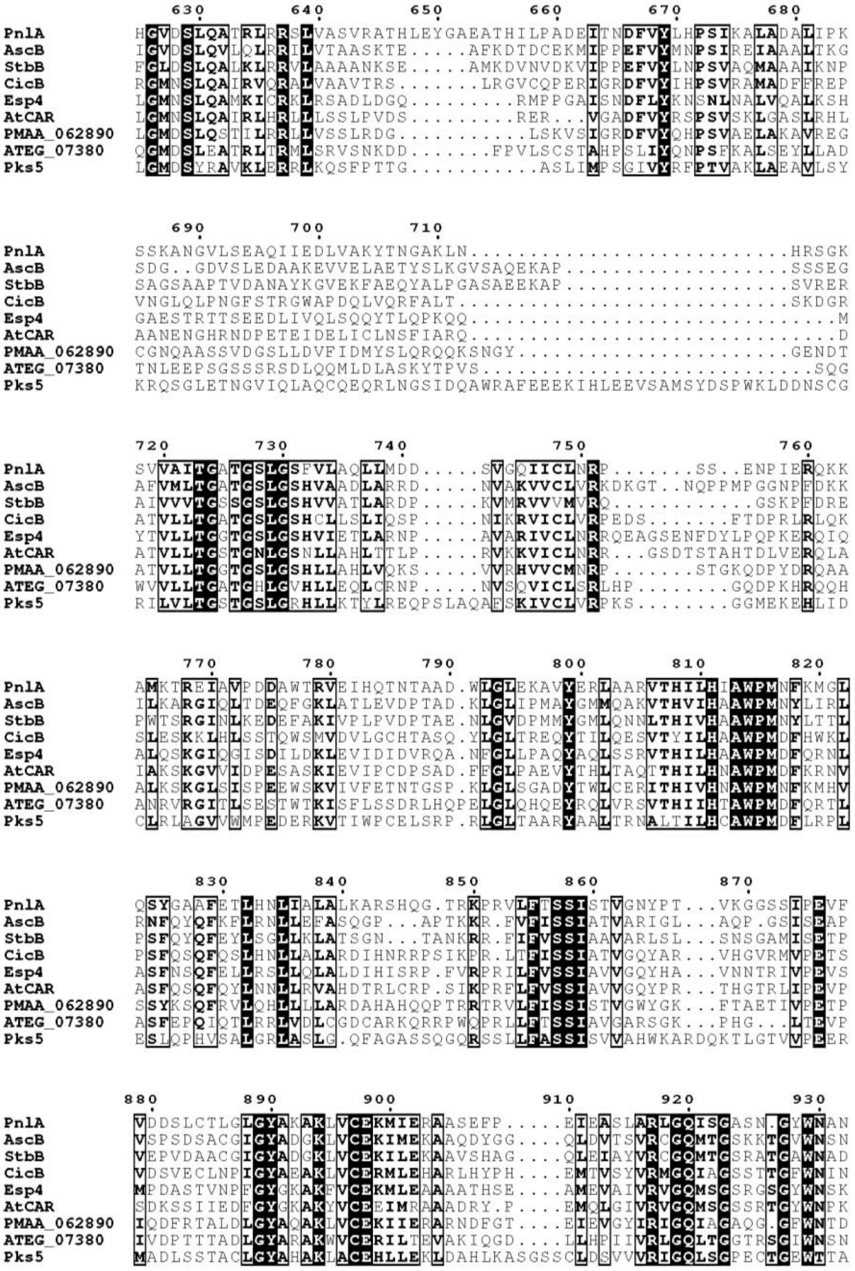

(*continued*)

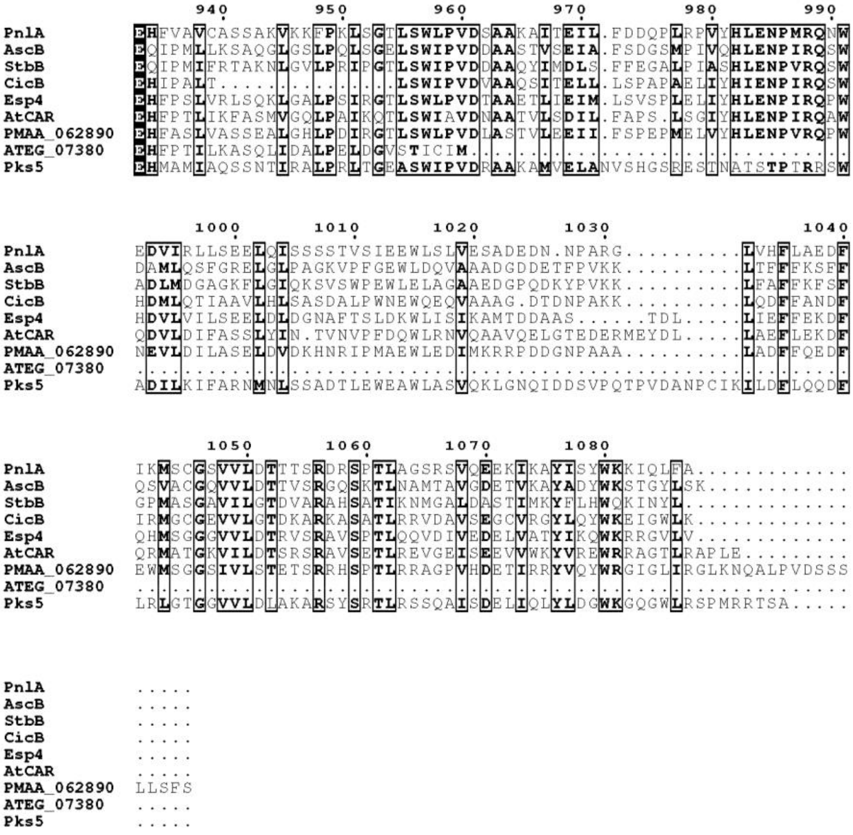

Figure S5. Alignment of fungal CARs located in clade VI, including PnlA, StbB [5], AscB [6], CicB [7], Esp4 [8], AtCAR [9], PMAA_062890, ATEG_07380 and Pks5 [10].

References

1. Wang, X.; Zhang, X.; Liu, L.; Xiang, M.; Wang, W.; Sun, X.; Che, Y.; Guo, L.; Liu, G.; Guo, L.; et al. Genomic and transcriptomic analysis of the endophytic fungus *Pestalotiopsis fici* reveals its lifestyle and high potential for synthesis of natural products. *BMC Genomics.* **2015**, *16*, 28, doi:10.1186/s12864-014-1190-9.
2. Sommers, C.H.; Rajkowski, K.T. Inactivation of *Escherichia coli* JM109, DH5α, and O157:H7 suspended in Butterfield's phosphate buffer by gamma irradiation. *J. Food Sci.* **2008**, *73*, M87-M90, doi:10.1111/j.1750-3841.2007.00647.x.
3. Ma, S.M.; Li, J.W.; Choi, J.W.; Zhou, H.; Lee, K.K.; Moorthie, V.A.; Xie, X.; Kealey, J.T.; Da Silva, N.A.; Vederas, J.C.; et al. Complete reconstitution of a highly reducing iterative polyketide synthase. *Science* **2009**, *326*, 589-592, doi:10.1126/science.1175602.
4. Zou, Y.; Garcia-Borràs, M.; Tang, M.C.; Hirayama, Y.; Li, D.H.; Li, L.; Watanabe, K.; Houk, K.N.; Tang, Y. Enzyme-catalyzed cationic epoxide rearrangements in quinolone alkaloid biosynthesis. *Nat. Chem. Biol.* **2017**, *13*, 325-332, doi:10.1038/nchembio.2283
5. Li, C.; Matsuda, Y.; Gao, H.; Hu, D.; Yao, X.S.; Abe, I. Biosynthesis of LL-Z1272β: discovery of a new member of NRPS-like enzymes for aryl-aldehyde formation. *ChemBioChem* **2016**, *17*, 904-907, doi:10.1002/cbic.201600087.
6. Araki, Y.; Awakawa, T.; Matsuzaki, M.; Cho, R.; Matsuda, Y.; Hoshino, S.; Shinohara, Y.; Yamamoto, M.; Kido, Y.; Inaoka, D.K.; et al. Complete biosynthetic pathways of ascofuranone and ascochlorin in *Acremonium egyptiacum*. *Proc. Natl. Acad. Sci. U.S.A.* **2019**, *116*, 8269-8274, doi:10.1073/pnas.1819254116.
7. Sanchez, J.F.; Entwistle, R.; Corcoran, D.; Oakley, B.R.; Wang, C.C.C. Identification and molecular genetic analysis of the cichorine gene cluster in *Aspergillus nidulans*. *MedChemComm* **2012**, *3*, 997-1002, doi:10.1039/C2MD20055D.
8. Zhu, G.; Hou, C.; Yuan, W.; Wang, Z.; Zhang, J.; Jiang, L.; Karthik, L.; Li, B.; Ren, B.; Lv, K.; et al. Molecular networking assisted discovery and biosynthesis elucidation of the antimicrobial spiroketals epicospirocins. *Chem. Commun.* **2020**, *56*, 10171-10174, doi:10.1039/D0CC03990J.
9. Wang, M.; Zhao, H. Characterization and engineering of the adenylation domain of a NRPS-like protein: A potential biocatalyst for aldehyde generation. *ACS Catal.* **2014**, *4*, 1219-1225, doi:10.1021/cs500039v.
10. Reyes-Fernández, E.Z.; Shi, Y.-M.; Grün, P.; Bode, H.B.; Bölker, M. An unconventional melanin biosynthesis pathway in *Ustilago maydis*. *Appl. Environ. Microbiol.* **2021**, *87*, e01510-e01520, doi:10.1128/AEM.01510-20.
